# Abnormal Magnetic Field Effects on Electrogenerated Chemiluminescence

**DOI:** 10.1038/srep09105

**Published:** 2015-03-16

**Authors:** Haiping Pan, Yan Shen, Hongfeng Wang, Lei He, Bin Hu

**Affiliations:** 1 Wuhan National Laboratory for Optoelectronics and School of Optical and Electronic Information, Huazhong University of Science and Technology, Wuhan 430074, China; 2 Department of Materials Science and Engineering, University of Tennessee, Knoxville, TN 37996, USA

## Abstract

We report abnormal magnetic field effects on electrogenerated chemiluminescence (MFE_ECL_) based on triplet emission from the Ru(bpy)_3_Cl_2_-TPrA electrochemical system: the appearance of MFE_ECL_ after magnetic field ceases. In early studies the normal MFE_ECL_ have been observed from electrochemical systems during the application of magnetic field. Here, the abnormal MFE_ECL_ suggest that the activated charge-transfer [Ru(bpy)_3_^3+^ … TPrA^•^] complexes may become magnetized in magnetic field and experience a long magnetic relaxation after removing magnetic field. Our analysis indicates that the magnetic relaxation can gradually increase the density of charge-transfer complexes within reaction region due to decayed magnetic interactions, leading to a positive component in the abnormal MFE_ECL_. On the other hand, the magnetic relaxation facilitates an inverse conversion from triplets to singlets within charge-transfer complexes. The inverse triplet → singlet conversion reduces the density of triplet light-emitting states through charge-transfer complexes and gives rise to a negative component in the abnormal MFE_ECL_. The combination of positive and negative components can essentially lead to a non-monotonic profile in the abnormal MFE_ECL_ after ceasing magnetic field. Nevertheless, our experimental studies may reveal un-usual magnetic behaviors with long magnetic relaxation from the activated charge-transfer [Ru(bpy)_3_^3+^ … TPrA^•^] complexes in solution at room temperature.

The magnetic field effects on electrogenerated chemiluminescence intensity, known as MFE_ECL_, have been intensively used to investigate the spin-dependent reaction routes in electrochemical reactions^1^,^2^,^3^. The most important step of generating electrogenerated chemiluminescence (ECL) can be formulated as the inter-molecular electron transfer between strong reductant A^−^ and oxidant D^+^ (A^−^ + D^+^ → A* + D) in electrochemical reactions^4^,^5^. In general, the MFE_ECL_ can be generated through two different channels, the so-called density and conversion channels, as shown in Fig. 1a. In the density channel, a magnetic field can change the density of light-emitting states by exerting Lorentz and magnetizing forces on reactant radicals (A^−^ and D^+^) and altering the total density of reactants in the reaction zone, generating density-based MFE_ECL_^6^. In the conversion channel, the conversion between singlets and triplets in both intermediate charge-transfer [A^−^ … D^+^] complexes and light emitting states may be modified by an applied magnetic field through spin mixing^3^,^4^,^7^, leading to conversion-based MFE_ECL_. It should be noted that the density-based MFE_ECL_ usually occurs on intermediate activated charge-transfer [A^−^ … D^+^] complexes, regarded as the precursor to the light-emitting states, because the Lorentz and magnetizing forces can be normally applied onto radical ions. Essentially, the change in the density of charge-transfer [A^−^ … D^+^] complexes can lead to a modification on the concentration of light-emitting states (A*) within reaction zone for the development of density-based MFE_ECL_. On the other hand, the conversion-based MFE_ECL_ can be, in principle, generated through both charge-transfer complexes and light-emitting states. In charge-transfer complexes a magnetic field can conveniently change the conversion between singlets and triplets by modifying the singlet-triplet intersystem crossing through spin mixing, eventually changing the singlet and triplet populations of light-emitting states^4^. In light-emitting states, an external magnetic field is not able to disturb the singlet-triplet intersystem crossing due to the strong spin-exchange interaction^8^ and spin-orbital coupling within the Ru(bpy)_3_^2+^* 9 in our system. But an applied magnetic field can change triplet-triplet annihilation^1^,^2^,^10^,^11^ and triplet-charge reaction^6^,^12^ in light-emitting states. This can cause a change on the population of light-emitting triplets for the development of conversion-based MFE_ECL_. Therefore, the conversion-based MFE_ECL_ can involve in both charge-transfer complexes and light-emitting states including (i) the conversion between singlets and triplets in charge-transfer complexes, and (ii) triplet-triplet annihilation and triplet-charge reaction in light-emitting states. However, it has been shown that the conversion in charge-transfer complexes is a dominant component in the conversion-based MFE_ECL_^13^,^14^. As a result, the overall MFE_ECL_ can be mainly generated by magnetically changing the density of reactants through Lorentz force and magnetizing force effects and the conversion between singlets and triplets in charge-transfer complexes through spin mixing in electrochemical systems, as schematically shown in Fig. 1a.

In this work, we report abnormal MFE_ECL_ in the Ru(bpy)_3_Cl_2_-TPrA electrochemical system and explore the possible magnetization of charge-transfer [Ru(bpy)_3_^3+^ … TPrA^•^] complexes within reaction zone. The ECL system used in our investigations contains tris(2,2′-bipyridine) ruthenium(II) (Ru(bpy)_3_^2+^) and tertiary amines as coreactants. Here we choose tripropylamine (TPrA) because among many coreactants used in Ru(bpy)_3_^2+^ based ECL systems TPrA appears to produce the highest light levels^15^. The universal ECL reaction routes of Ru(bpy)_3_^2+^ with tertiary amines can be expressed as Fig. 1b^16^,^17^.

We can see from Fig. 1b that the electro-oxidation of TPrA generates a positively charged radical ion TPrA^+•^. Then the α-carbon of TPrA^+•^ rapidly deprotonates to generate TPrA^•^, which can be paired with the Ru(bpy)_3_^3+^ to form activated charge-transfer [Ru(bpy)_3_^3+^ … TPrA^•^] complexes for generating the light-emitting Ru(bpy)_3_^2+^*. It should be noted that the light emission is from the triplet Ru(bpy)_3_^2+^* which is controlled by the electron transfer process between activated radicals Ru(bpy)_3_^3+^ and TPrA^•^ within the charge-transfer [Ru(bpy)_3_^3+^ … TPrA^•^] complexes while the electrical current is governed only by the electro-oxidation and deprotonation of TPrA. Therefore, magnetic field effects on ECL intensity (MFE_ECL_) and electrical current (MC) can be used to distinguish the critical spin-dependent processes in this electrochemical system.

## Results and Discussion

**Figure 2:**
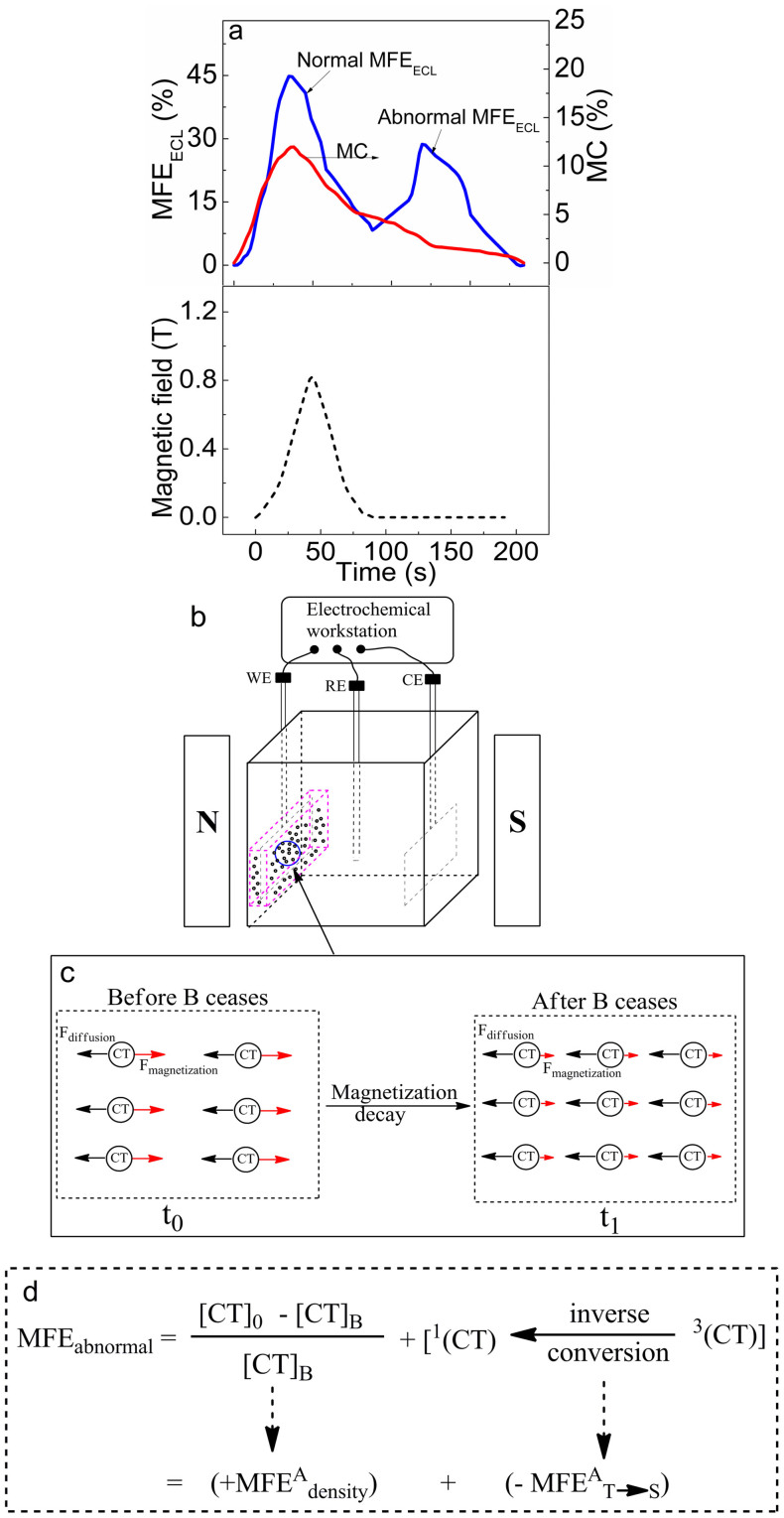
(a) MFE_ECL_ and MC are shown at a constant electrode-potential of 1.28 V rapidly changed from low 1.11 V. (b) Experimental setup for MFE_ECL_/MC measurements by placing an electrochemical cell in a magnetic field. (c) Activated charge-transfer (CT) [Ru(bpy)_3_^3+^ … TPrA^•^] complexes are subject to both diffusion and magnetizing forces in reaction zone. Removing an external magnetic field can break the previously established equilibrium on the CT density, consequently leading to an increase on the mass transport of reactants with the consequence of increasing the CT density. (d) Schematic diagram to show the generation of abnormal MFE_ECL_ through density and conversion channels. [CT]_B_ and [CT]_0_ are the densities of charge-transfer [Ru(bpy)_3_^3+^ … TPrA^•^] complexes upon applying and removing a magnetic field.

Fig. 2a shows the MFE_ECL_ and MC generated from the electrochemical Ru(bpy)_3_Cl_2_-TPrA system. The observed magnetic field effects can be divided into two regimes: during the application of magnetic field and after ceasing magnetic field. In normal regime, both MFE_ECL_ and MC signals follow the profile of magnetic field, generating positive magnetic field effects. However, in abnormal regime the two signals show different behaviors: the MFE_ECL_ signal increases to reach a second peak and then gradually decays to zero while the MC signal gradually decreases to zero, after the applied magnetic field ceases. Usually, the magnetic field effects only appear during the application of a magnetic field^1^,^2^,^3^,^7^,^13^, which are named as normal MFE_ECL_ and MC. Here, we can see from Fig. 2a that the MFE_ECL_ show an abnormal phenomenon: light intensity increases and then decreases after ceasing magnetic field at a constant electrochemical condition. We name this phenomenon as abnormal MFE_ECL_. To our best knowledge, it is the first time to report abnormal MFE_ECL_ after an applied magnetic field is removed. It is obvious that in abnormal regime the MFE_ECL_ and MC show very distinct behaviors with significant and negligible values, respectively (Fig. 2a). This distinct difference indicates that the MFE_ECL_ and MC can be attributed to charge-transfer [Ru(bpy)_3_^3+^ … TPrA^•^] complexes and TPrA^•^, separately. The abnormal MFE_ECL_ may imply that the intermediate activated charge-transfer [Ru(bpy)_3_^3+^ … TPrA^•^] complexes still experience magnetic interactions after applied magnetic field is removed, which changes the concentration of light-emitting states within reaction zone. Based on this analysis, we can suggest that the charge-transfer [Ru(bpy)_3_^3+^ … TPrA^•^] complexes may become magnetized during the application of magnetic field and then undergo a long magnetic relaxation after applied magnetic field ceases. Generally, the magnetic relaxation after ceasing magnetic field can affect both the density of charge-transfer complexes and the conversion between singlets and triplets within charge-transfer complexes, generating abnormal MFE_ECL_ (Fig. 2d). First, upon ceasing magnetic field the magnetic relaxation can gradually decrease the magnetic interaction between the charge-transfer complexes, disturbing the density equilibrium within reaction zone previously established by the competition between magnetizing force and diffusion force during the application of magnetic field (Fig. 2c). Disturbing the density equilibrium can increase the mass transport of reactants into the reaction zone with the consequence of increasing electrochemical reaction, leading to a positive component in abnormal MFE_ECL_ (+MFE^A^_density_) through density channel. Second, the magnetic relaxation after ceasing magnetic field can cause spin dephasing within charge-transfer complexes and consequently generates an inverse conversion from triplets to singlets in charge-transfer complexes, decreasing the population of triplet light-emitting states and generating a negative component in abnormal MFE_ECL_ (−MFE^A^_T→S_) through conversion channel. Therefore, the density-based +MFE^A^_density_ and conversion-based −MFE^A^_T→S_ generate the non-monotonic curve of abnormal MFE_ECL_.

**Figure 3:**
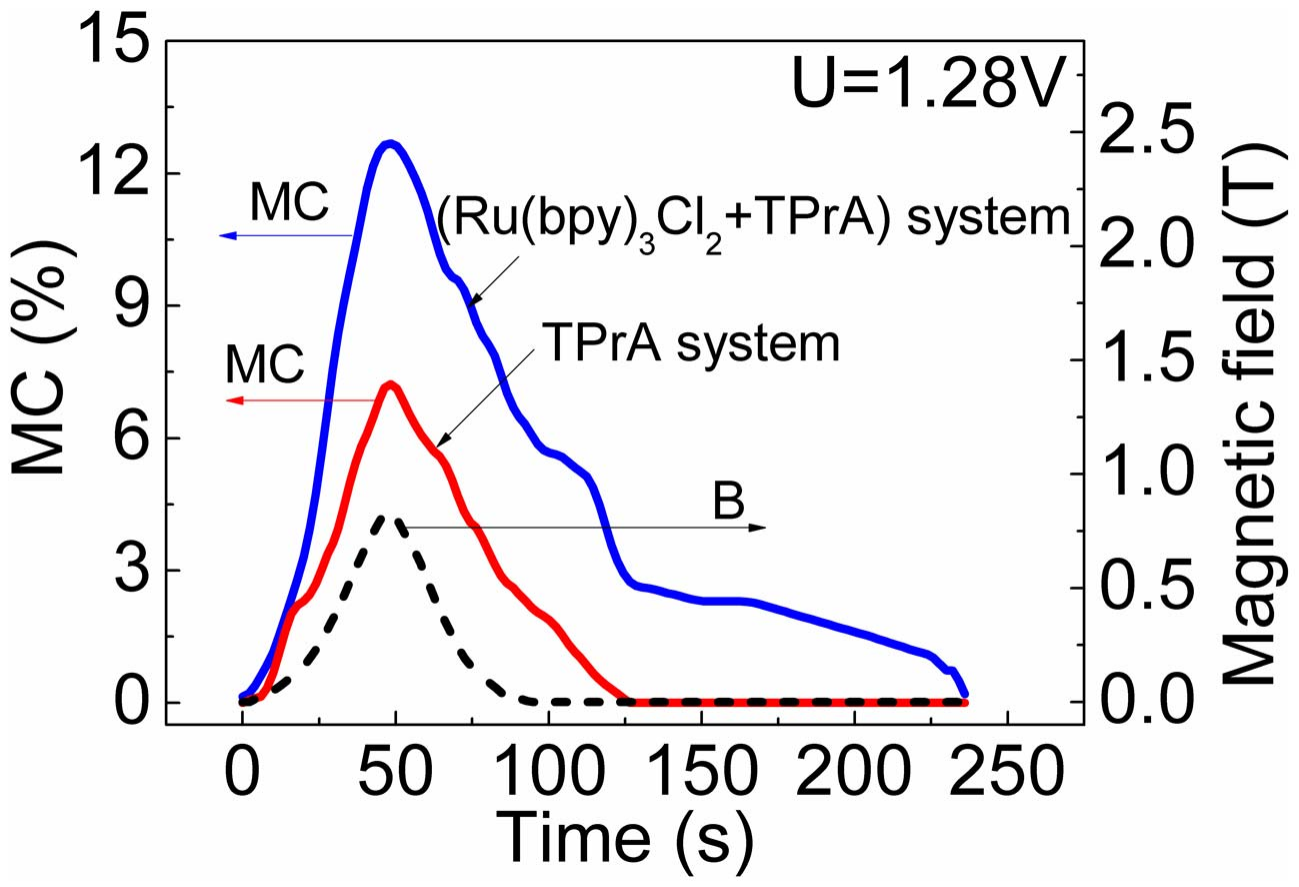
MC from two different electrochemical systems: (Ru(bpy)_3_Cl_2_ + TPrA) and TPrA with the same applied electrode potential of 1.28 V. The two systems both use sodium dihydrogen phosphate as supporting electrolyte. The (Ru(bpy)_3_Cl_2_ + TPrA) system contains 1 mM Ru(bpy)_3_Cl_2_ and 0.08 M TPrA as reactants. The TPrA system contains only 0.08 M TPrA as reactant.

We should note that only normal MC is observed in our system. Because MC is generated by Lorentz force or spin polarization effects. Here, our measurements were performed at the zero angle condition between magnetic field and charge transport (Fig. 2b). Additionally, the observed MC does not show appreciable angle dependence when the angle between magnetic field and charge transport is changed. Thus Lorentz force can be neglected in our system. This leaves the spin polarization effects responsible for the observed MC. In principle, spin polarization can increase the formation of TPrA^•^ radicals by decreasing the C-H bond recovery through spin configuration effects^13^ in electrochemical reaction. This can cause an increase on the oxidation rate of TPrA on working electrode, leading to an increase on electrical current upon applying a magnetic field. Therefore, the spin polarization effects of TPrA^•^ radicals can generate a positive MC. Clearly, the MC results (Fig. 3) confirm that the TPrA^•^ radicals are spin polarized in a magnetic field. In particular, the spin polarization of TPrA^•^ radicals provides a pre-condition for the charge-transfer [Ru(bpy)_3_^3+^ … TPrA^•^] complexes to become magnetized with abnormal MFE_ECL_ in the electrochemical system.

Now we discuss the possible mechanism to generate the magnetized charge-transfer [Ru(bpy)_3_^3+^ … TPrA^•^] complexes. It is noted that the distance between the oxidant Ru(bpy)_3_^3+^ and reductant TPrA^•^ during the electron transfer process is usually in the range of 4–6 Å 4. Thus, the [Ru(bpy)_3_^3+^ … TPrA^•^] complexes are often defined as activated charge-transfer complexes. In this situation, the molecular orbitals may overlap to generate the intermolecular contacts between the nitrogen p orbitals of TPrA^•^ and the bpy π orbitals of Ru(bpy)_3_^3+^ in charge-transfer [Ru(bpy)_3_^3+^ … TPrA^•^] complexes. It has been found that the overlap of molecular orbitals in several stacking modes of allyl and nitroxide radical systems play an essential role in generating ferromagnetic interaction in extended molecular systems^18^,^19^,^20^. The literature work has also found that the intermolecular contacts between the NO groups and the phenyl ring may also assist the ferromagnetic interaction in spin radicals. Therefore, we can expect a possible ferromagnetic interaction in our charge-transfer [Ru(bpy)_3_^3+^ … TPrA^•^] complexes in an applied magnetic field. In addition, it has been shown that the ferromagnetic interaction can possibly occur between charge-transfer complexes due to the overlap of spatially extended wavefunctions^21^,^22^,^23^. As a result, the charge-transfer [Ru(bpy)_3_^3+^ … TPrA^•^] complexes can be comparable to the nitronyl nitroxide radicals which exhibit intermolecular ferromagnetic interaction due to the overlap of partially occupied molecular orbitals^18^,^24^,^25^,^26^. It should be noted that the exchange coupling between the nitrogen p orbitals of TPrA^•^ and the bpy π orbitals of Ru(bpy)_3_^3+^ can be treated as a hybridization between Ru(bpy)_3_^3+^ and TPrA^•^. This hybridization is similar to the exchange coupling between a π-conjugated orbitals on the quinoline rings and nitrogen p_z_ orbitals which generates ferromagnetic interaction between the spin moments^27^,^28^,^29^,^30^,^31^,^32^. Therefore, the activated charge-transfer [Ru(bpy)_3_^3+^ … TPrA^•^] complexes may be magnetized due to (i) spin interactions of Ru(bpy)_3_^3+^ and TPrA^•^ radical ions and (ii) magnetic coupling between the charge-transfer [Ru(bpy)_3_^3+^ … TPrA^•^] complexes.

We should note that it is rare to observe magnetic interaction in charge-transfer complexes at room temperature. However, for some specific situation, magnetic coupling between two radicals which is connected to the same metal center can be obtained, forming molecular based ferromagnets at room temperature^33^,^34^. In another case, a spontaneous magnetization can be induced from the exchange interactions between the localized spins on metal ion and the spins on organic radicals^35^. In our ECL system, the possible magnetic coupling between [Ru(bpy)_3_^3+^ … TPrA^•^] complexes can be induced by (i) the spin interaction between Ru(bpy)_3_^3+^ and TPrA^•^ radicals due to the overlap between molecular orbitals of Ru(bpy)_3_^3+^ and TPrA^•^ within the charge-transfer complexes, (ii) the electron transfer from the nitrogen p orbitals of TPrA^•^ to the bpy π orbitals of Ru(bpy)_3_^3+^ and (iii) the orbital overlap between the charge-transfer complexes.

**Figure 4:**
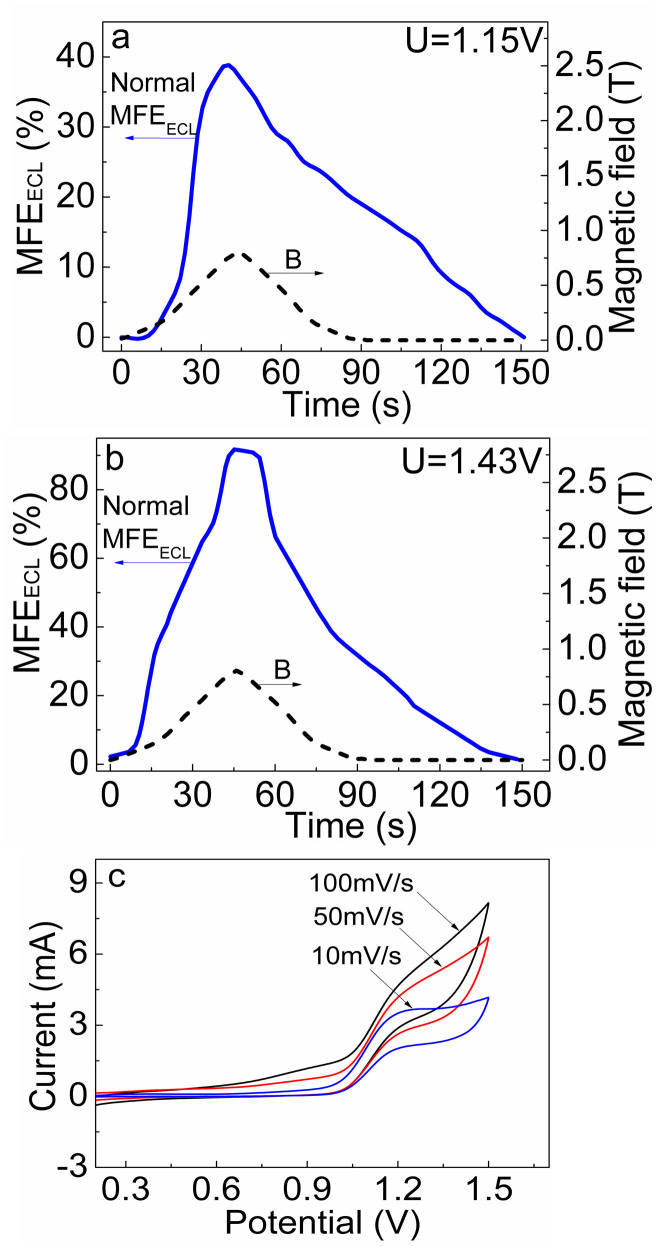
Only normal MFE_ECL_ can be observed by slowly increasing electrode potential in the electrochemical (Ru(bpy)_3_Cl_2_ + TPrA) system. (a) Electrode potential was set at 1.15 V. (b) Electrode potential was set 1.43 V. (c) Cyclic voltammograms collected at different scanning rates.

Next we discuss the critical parameters that can be accountable for magnetized charge-transfer [Ru(bpy)_3_^3+^ … TPrA^•^] complexes. We can see in Fig. 4a and 4b that the abnormal MFE_ECL_ are largely reduced when the electrode potential is slowly increased between selected potentials of 1.15 V and 1.43 V. In our system, the magnetic coupling require a high density of activated triplet charge-transfer complexes (with spin S = 1). Obviously, the largely reduced abnormal MFE_ECL_ upon slowly increasing electrical potential indicate the low density of charge-transfer [Ru(bpy)_3_^3+^ … TPrA^•^] complexes. This argument is made based on the following consideration. In general, a low electrode potential can lead to a low oxidation rate of reactants at the working electrode. In this case, the quantity of intermediate active radicals of Ru(bpy)_3_^3+^ and TPrA^•^ generated on the working electrode surface is low, diminishing the magnetic coupling. When the electrode potential is slowly increased to a high value, the reactants (mainly TPrA) are continuously consumed during the electrochemical process, as indicated by the little change in the oxidation current when applied electrode potential increases at slow-scanning rate of 10 mV/s (Fig. 4c). In this case, at high electrode potential the quantity of oxidation products (mainly TPrA^•^) is still not high, leading to a low density of charge-transfer complexes and a negligible magnetic coupling between the activated charge-transfer complexes. In our experiments, the abnormal MFE_ECL_ can only be clearly observed when electrode potential rapidly changes from low to high value. In this case, there are a large amount of active radicals Ru(bpy)_3_^3+^ and TPrA^•^ generated nearby the working electrode at a short period of time, as indicated by the rapid enhancement of oxidation current as applied electrode potential increases at fast-scanning rate of 100 mV/s (Fig. 4c). This condition can lead to a high density of activated charge-transfer [Ru(bpy)_3_^3+^ … TPrA^•^] complexes with magnetic coupling within the reaction zone to generate the abnormal MFE_ECL_. Therefore, we can confirm that the high densities of both Ru(bpy)_3_^3+^ and TPrA^•^ in the reaction zone form a necessary condition to generate a strong magnetic coupling between the charge-transfer [Ru(bpy)_3_^3+^ … TPrA^•^] complexes. Essentially, strong magnetic coupling between the charge-transfer [Ru(bpy)_3_^3+^ … TPrA^•^] complexes generates magnetized charge-transfer [Ru(bpy)_3_^3+^ … TPrA^•^] complexes, leading to abnormal MFE_ECL_. Furthermore, in order to observe the significant abnormal MFE_ECL_, 1 mM Ru(bpy)_3_^2+^ is enough but the concentration of TPrA should be at least 0.08 M or higher.

**Figure 5:**
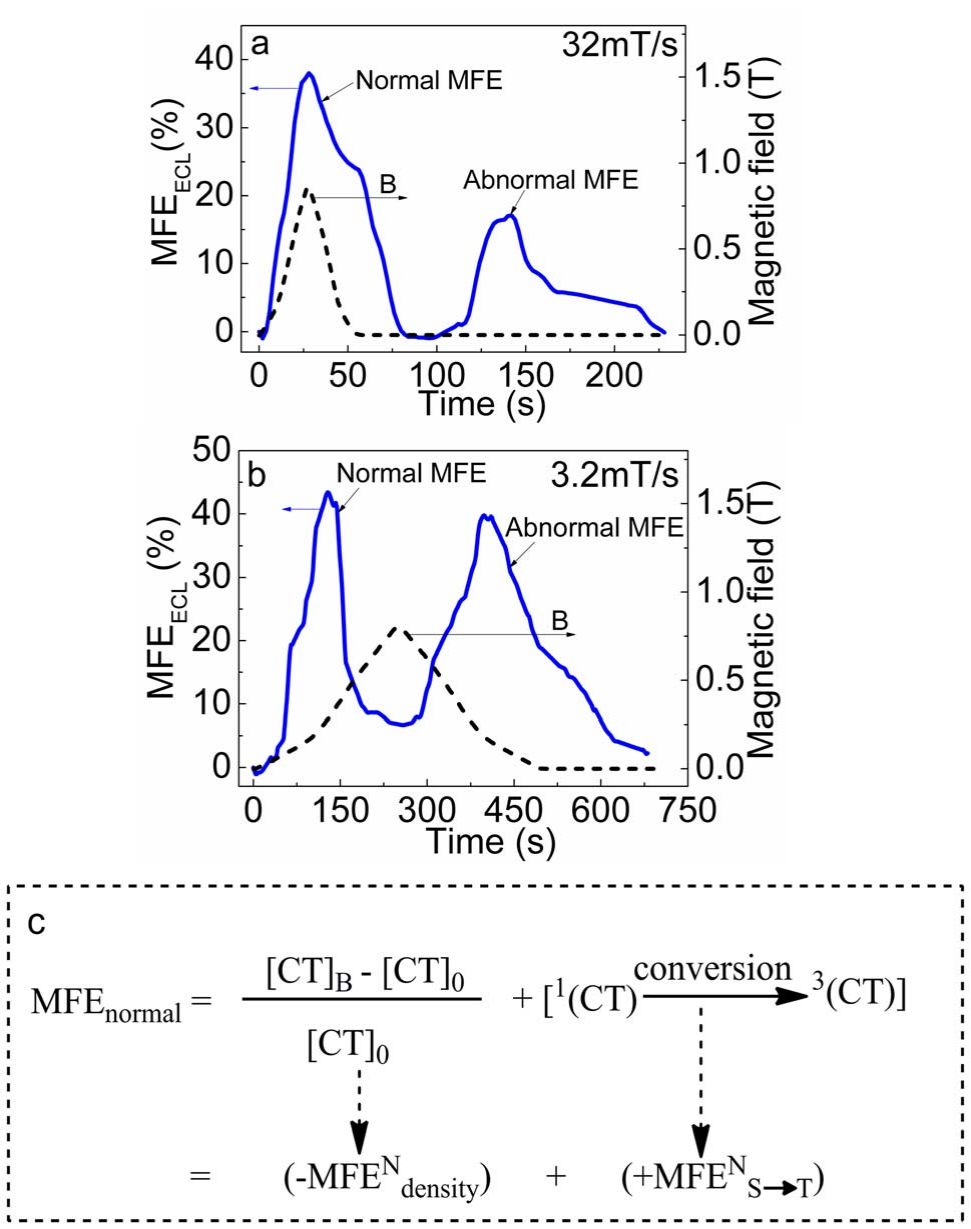
Normal and abnormal MFE_ECL_ from the electrochemical (Ru(bpy)_3_Cl_2_ + TPrA) system under different field-sweeping rates at the electrode potential of 1.28 V. (a) Fast field-sweeping rate = 32 mT/s; (b) Slow field-sweeping rate = 3.2 mT/s. (c) Schematic diagram to show the generation of normal MFE_ECL_ through density and conversion channels. [CT]_0_ and [CT]_B_ are the densities of charge-transfer complex [Ru(bpy)_3_^3+^ … TPrA^•^] before and during the application of a magnetic field.

Here we consider additional experimental evidence to support the possible magnetized charge-transfer [Ru(bpy)_3_^3+^ … TPrA^•^] complexes. Fig. 5a and 5b shows both normal and abnormal MFE_ECL_ at fast sweeping (32 mT/s) and slow sweeping (3.2 mT/s) rates. For the fast sweeping rate, the normal MFE_ECL_ peak (37%) coincides with the maximal value of applied magnetic field. However, the abnormal MFE_ECL_ peak is decreased to 17%. For a slow sweeping rate, the peak (43%) of normal MFE_ECL_ appears before the maximum of applied magnetic field. The abnormal MFE_ECL_ peak remains at a high value (40%). The sweeping rate effects on both normal and abnormal MFE_ECL_ essentially reflect the possible magnetization response time during the application of magnetic field and the magnetic relaxation after the magnetic field is removed. During the application of magnetic field, the induced magnetization can decrease the density of charge-transfer [Ru(bpy)_3_^3+^ … TPrA^•^] complexes through repulsive magnetic interactions but increases the conversion from singlets to triplets within charge-transfer [Ru(bpy)_3_^3+^ … TPrA^•^] complexes, as indicated in Fig. 5c. In addition, the density and conversion-based MFE_ECL_ have negative and positive signs in normal regime, respectively. From the observed positive normal MFE_ECL_ we can suggest that the singlet → triplet conversion is a major process at fast sweeping rate. Specifically, at fast sweeping rate the magnetic field can increase the triplet density of light-emitting states Ru(bpy)_3_^2+^* by increasing the singlet → triplet conversion through spin polarization effects in the charge-transfer [Ru(bpy)_3_^3+^ … TPrA^•^] complexes, generating a positive conversion-based component (+MFE^N^_S→T_) in normal regime. Furthermore, during the fast sweeping the density of charge-transfer complexes does not have sufficient time to vary within reaction zone, leading to a negligible negative density-based normal component (−MFE^N^_density_). However, in abnormal regime both conversion and density-based MFE_ECL_ are slow due to the long magnetic relaxation of charge-transfer [Ru(bpy)_3_^3+^ … TPrA^•^] complexes. In addition, a fast sweeping rate may lead to a lower spin polarization probably due to in-sufficient magnetization response time in the charge-transfer [Ru(bpy)_3_^3+^ … TPrA^•^] complexes. This can essentially generate a lower value for abnormal MFE_ECL_ after ceasing magnetic field, as shown in Fig. 5a. A slow sweeping rate may give a higher spin polarization in the charge-transfer [Ru(bpy)_3_^3+^ … TPrA^•^] complexes. This can give rise to a higher value for abnormal MFE_ECL_, as indicated in Fig. 5b. A higher spin polarization can also give a larger singlet → triplet conversion component towards the triplet formation of light-emitting states in the normal MFE_ECL_. On the other hand, with a slow sweeping rate the singlet → triplet conversion component can quickly reach its maximum even prior to the maximum of magnetic field in normal regime. However, with a slow sweeping rate the density component may be slowly activated with the negative sign after the fast singlet → triplet conversion is finished in the normal regime. The combination of the fast singlet → triplet conversion-based component with positive sign and the slow density-based component with negative sign can lead to the normal MFE_ECL_ with its peak value appeared before the maximum of magnetic field. Nevertheless, the sweeping rate effects of both normal and abnormal MFE_ECL_ provide further experimental evidence to confirm that the charge-transfer [Ru(bpy)_3_^3+^ … TPrA^•^] complexes may be magnetized in magnetic field and experience a long relaxation after magnetic field is removed.

## Conclusion

Based on the Ru(bpy)_3_Cl_2_-TPrA electrochemical system, we observe not only normal MFE_ECL_ during the application of magnetic field but also abnormal MFE_ECL_ after applied magnetic field ceases. The abnormal MFE_ECL_ suggests that the charge-transfer [Ru(bpy)_3_^3+^ … TPrA^•^] complexes may become magnetized in magnetic field and then experience a long magnetic relaxation after magnetic field is removed. On the other hand, we observe a negligible abnormal MC from this system after removing magnetic field. The distinct behavior between MFE_ECL_ and MC in abnormal regime confirm that the charge-transfer [Ru(bpy)_3_^3+^ … TPrA^•^] complexes are magnetized species responsible for the abnormal MFE_ECL_ in the electrochemical system. The magnetic relaxation after ceasing magnetic field generates two opposite effects: increasing the density of charge-transfer complexes due to decayed repulsive magnetic interactions and inducing an inverse triplet → singlet conversion due to gradually relaxed spin alignments in charge-transfer [Ru(bpy)_3_^3+^ … TPrA^•^] complexes. The former and latter generate the density-based MFE_density_ with positive sign and the conversion-based MFE_conversion_ with negative sign in abnormal regime, respectively, after removing magnetic field. Clearly, our experimental studies on abnormal MFE may reveal magnetic coupling between intermediate activated charge-transfer [Ru(bpy)_3_^3+^ … TPrA^•^] complexes with long magnetic relaxation in solution. The magnetic behavior can be attributed to the overlap of partially occupied orbitals initiated by electron transfer within the charge-transfer [Ru(bpy)_3_^3+^ … TPrA^•^] complexes and the wavefunction overlaps between the charge-transfer [Ru(bpy)_3_^3+^ … TPrA^•^] complexes at high density within reaction zone.

## Methods

Chemical tris(2,2′-bipyridine)ruthenium(II) dichioride(Ru(bpy)_3_Cl_2_), tripropylamine(TPrA), and sodium dihydrogen phosphate(NaH_2_PO_4_) were all purchased from Aldrich and used as received. The ECL solution concentration were 1 mM Ru(bpy)_3_^2+^/0.08 M TPrA/0.1 M sodium dihydrogen phosphate in de-ionized water, 0.08 M TPrA/0.1 M sodium dihydrogen phosphate in de-ionized water. All solutions were deoxygenated by bubbling with nitrogen for at least 15 min before the measurement. Electrochemical cell was designed as follows: two flat Pt foil plate electrodes (10 mm × 15 mm × 0.3 mm) served as the working electrode and counting electrode; a silver chloride (Ag/AgCl) electrode served as reference electrode. Electrochemical cell was placed in a magnetic field generated by an electromagnet. Cyclic voltammetry was carried out and electrical current was recorded with the model CHI 750D electrochemical workstation. The electrogenerated chemiluminescence spectrum and intensity were characterized by FLS920 Fluorescence Spectrometer (Edinburgh Instrument) equipped with an optical fiber connection. MFE_ECL_ and magnetocurrent (MC) are defined as relative electrogenerated chemiluminescence intensity and current changes caused by an applied magnetic field (MFE = (S_B_ − S_0_)/S_0_ × 100%), where S_B_ and S_0_ are the signal intensities with and without a magnetic field).

## Author Contributions

H.P. did the experimental measurements. B.H. guided the research. B.H. and H.P. wrote the main manuscript text. Y.S., H.W. and L.H. revised the manuscript. All authors contributed to data analysis and discussions.

## Figures and Tables

**Figure 1:**
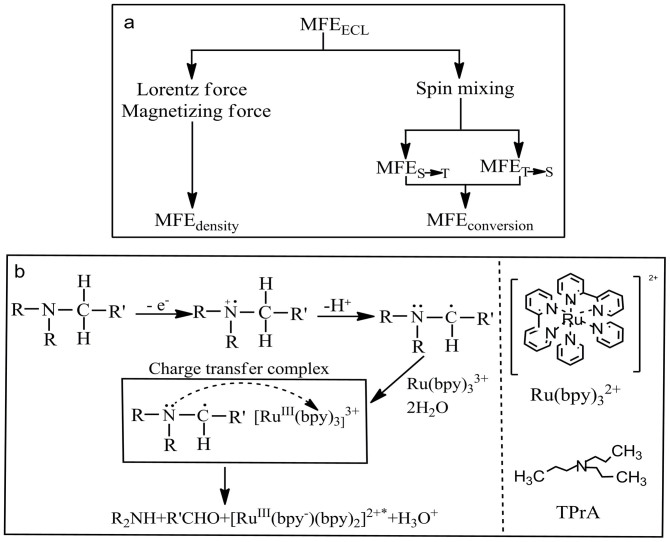
(a) MFE_ECL_ are generated by density and conversion channels due to Lorentz and magnetizing force exerting on magnetized activated [A^−^ … D^+^] complexes and spin mixing between singlet ^1^[A^−^ … D^+^] and triplet ^3^[A^−^ … D^+^] complexes, respectively. (b) The reaction routes are shown for the formation of charge-transfer [Ru(bpy)_3_^3+^ … TPrA^•^] complexes. The molecular structures are also shown for Ru(bpy)_3_^2+^ and TPrA. The R and R′ denote CH_2_CH_2_CH_3_ and CH_2_CH_3_.
